# Entropy-Driven Crystallization of Hard Colloidal Mixtures of Polymers and Monomers

**DOI:** 10.3390/polym16162311

**Published:** 2024-08-15

**Authors:** Olia Bouzid, Daniel Martínez-Fernández, Miguel Herranz, Nikos Ch. Karayiannis

**Affiliations:** Institute for Optoelectronic Systems and Microtechnology (ISOM) and Escuela Técnica Superior de Ingenieros Industriales (ETSII), Universidad Politécnica de Madrid (UPM), José Gutierrez Abascal 2, 28006 Madrid, Spain; olia.bouzid@alumnos.upm.es (O.B.); daniel.martinez.fernandez@upm.es (D.M.-F.); miguel.herranzf@upm.es (M.H.)

**Keywords:** Monte Carlo, athermal mixture, crystallization, entropy-driven phase transition, colloids, polymer, face-centered cubic, hexagonal close-packed, molecular simulation, dense packing, hard sphere, fivefold

## Abstract

The most trivial example of self-assembly is the entropy-driven crystallization of hard spheres. Past works have established the similarities and differences in the phase behavior of monomers and chains made of hard spheres. Inspired by the difference in the melting points of the pure components, we study, through Monte Carlo simulations, the phase behavior of athermal mixtures composed of fully flexible polymers and individual monomers of uniform size. We analyze how the relative number fraction and the packing density affect crystallization and the established ordered morphologies. As a first result, a more precise determination of the melting point for freely jointed chains of tangent hard spheres is extracted. A synergetic effect is observed in the crystallization leading to synchronous crystallization of the two species. Structural analysis of the resulting ordered morphologies shows perfect mixing and thus no phase separation. Due to the constraints imposed by chain connectivity, the local environment of the individual spheres, as quantified by the Voronoi polyhedron, is systematically more spherical and more symmetric compared to that of spheres belonging to chains. In turn, the local environment of the ordered phase is more symmetric and more spherical compared to that of the initial random packing, demonstrating the entropic origins of the phase transition. In general, increasing the polymer content reduces the degree of crystallinity and increases the melting point to higher volume fractions. According to the present findings, relative concentration is another determining factor in controlling the phase behavior of hard colloidal mixtures based on polymers.

## 1. Introduction

Self-assembly and self-organization are spontaneous processes, strongly related to thermal fluctuations, that dominate the behavior in a wide range of systems belonging to the general field of soft matter [1,2,3]. Phase transition [4] is perhaps the most common example of such processes. Packings composed of hard bodies are excellent case studies to better understand the complex phenomenon of crystallization, especially at the atomic level [5,6].

The most well-known example of entropy-driven phase transitions is the isotropic-to-nematic transition of hard rods, as originally predicted by Onsager [7], and verified by computer simulations, which demonstrated the formation of liquid crystals once specific conditions are met [8,9,10]. Another eminent example is the crystallization of hard spheres of uniform size in three dimensions, initially predicted by Kirkwood [11] and further confirmed by pioneering Molecular Dynamics (MD) [12] and Monte Carlo (MC) [13] simulations. The athermal nature of both systems leaves entropy as the sole factor that drives the behavior of the system. Thus, it is now well established that at sufficiently high concentrations and at constant volume, crystallization occurs because the final crystal, made of hard spheres, possesses more entropy than the initial amorphous packing [14,15]. For hard sphere monomers (*mon*) of the same diameter, initial estimates for the freezing and melting transitions were provided by Hoover and Ree [16] at $\varphi_{mon}^{F}\approx 0.494$ and $\varphi_{mon}^{M}\approx 0.545$, respectively, which have been revised to the more precise values of $\varphi_{mon}^{F}\approx 0.4915$ and $\varphi_{mon}^{M}\approx 0.5431$ by Vega and Noya [17]. From the thermodynamic perspective, all independent calculations converge to the fact that the face-centered cubic (FCC) crystal is only marginally more stable than the hexagonal close-packed (HCP) one, with the free-energy difference between them depending on the estimation method, the proximity (from the above) to the melting point, and the size of the system [18,19,20,21,22].

While the FCC crystal is the most stable polymorph and thus the one expected in experiments and simulations on hard-sphere crystallization, the most encountered configuration is a random hexagonal close-packed (rHCP) morphology with defects in the form of twins at the boundaries of the HCP and FCC domains [23,24]. Even if almost seven decades have passed since the first computational demonstration of hard sphere crystallization [25], crystal perfection in the form of a pure FCC crystal, with a minimal population of defects in the form of other crystallites or amorphous clusters, is very rarely reported. This is because the structural transition between polymorphs is too slow to be appropriately gauged in simulations and experiments, given the inherent limitations in the observation time.

Due to their simplicity, molecular simulations based on the hard-sphere model constitute an ideal tool to shed light on the mechanism of crystallization as they can assess the precursor structures, track the crystal nucleation and growth, and identify with precision the final ordered morphologies [24,26,27,28,29,30,31,32,33,34,35,36,37,38,39,40].

Athermal packings made of polymers possess specific challenges in their studies. Experimentally, despite the recent advances [41,42,43,44], it is significantly more difficult to synthesize hard colloidal polymers of precisely defined chain architecture and stiffness compared to monomeric analogs. Then, from the modeling perspective, the generation and equilibration of polymer-based systems can be a notoriously difficult simulation task, especially under extreme conditions of concentration or when very long and entangled chains are considered. A solution to this can be provided by MC schemes built around clever algorithms [45], like, for example, the end-bridging [46,47] and double-bridging [48,49] chain-connectivity-altering moves, originally developed for the atomistic simulation of poly(ethylene) melts. Inspired by the success at the atomistic level, simplified variants of the moves have been employed, which allowed the generation and equilibration of athermal packings of linear chains of tangent hard spheres even in the close vicinity of the maximally random jammed (MRJ) state [50] and under a wide variety of conditions, including extreme confinement [51,52,53,54]. In fact, it has been demonstrated that the performance of properly designed chain-connectivity-altering moves increases with volume fraction and reaches its maximum near the MRJ state [51].

With respect to the phase behavior of athermal polymer packings in the bulk, it has been demonstrated that linear, freely jointed chains of tangent hard spheres crystallize as monomers do [55,56,57]. According to the constant-volume simulations of Refs. [55,56], at packing densities *φ* ≤ 0.56, chain systems remain amorphous, while at *φ* = 0.58, crystallization occurs. This upper estimate for the phase transition of hard-sphere polymers (*pol*) has been recently revised in Ref. [58], according to which the melting point lies in the range $\varphi_{pol}^{M}\in (0.56,0.57]$. Further studies have demonstrated that chain length has a minimal effect on crystallization and the melting transition [55,56], while other factors like gaps in bonds [59,60] and chain stiffness [58,61,62] profoundly affect the phase behavior. Very long MC simulations have shown that athermal packings of very long chains (*N* = 1000, *N* being the molecular length) transit between different crystal polymorphs and eventually form a single FCC crystal of very high perfection [63]. Through semi-analytical calculations, it has been argued that, indeed, the FCC crystal is the thermodynamically most stable structure for athermal polymers [64], as is in the case of monomers [18,19,20,21,22].

The present work is motivated by comparing the underlying mechanisms for the crystallization of the pure-component systems: monomeric hard spheres and linear, freely jointed chains of tangent hard spheres. The similarities can be summarized as: (i) once a critical volume fraction is reached and given sufficient time, both systems crystallize [12,13,55,56,65]; (ii) an increase in the entropy drives the phase transition [55,63,64,66]; (iii) the local environment becomes more symmetric and spherical in the ordered phase [5,55,63]; and (iv) fivefold local symmetry acts as a competitor to the formation of close-packed crystallites [63,67,68]. Meanwhile, the differences are that (a) the melting point of athermal polymers [55,58] is shifted to higher packing densities compared to that of monomers [16,17] and (b) when tangency is enforced on bonded spheres, the resulting ordered morphologies show an rHCP layered morphology with a unique stacking direction, mostly free of structural defects in the form of fivefold local symmetry [69], while monomeric counterparts mainly form fivefold-ridden rHCP layers with multiple stacking directions [24,68].

Thus, we employ Monte Carlo simulations, executed through the Simu-D software (version 1.0) [54], to systematically explore the phase behavior of athermal mixtures made of fully flexible chains and individual monomers of identical diameters. Our objective is to study in detail the effect of the relative number fraction on the ability of the mixtures of polymer and monomers to crystallize in a concentration range above the melting point of monomers and below the one of polymers. The idea is to investigate whether the chains could act as inhibitors to the crystallization of monomers and/or whether monomers could act as promoters to the crystallization of chains. One should not discard further the possibility of phase separation splitting the system volume into polymer-rich and monomer-rich domains.

Towards this, we first provide an updated estimate of the melting point for freely jointed chains of tangent hard spheres. Then, we explore the phase behavior as a function of packing density and polymer content. In parallel, the ordered morphologies are gauged, and the level of mixing is quantified. Finally, the entropic origins of the spontaneous self-assembly are analyzed by comparing the shape measures of the Voronoi polyhedra between the initial random packing and the final crystal, further distinguishing between spheres belonging to chains and individual ones.

The paper is organized as follows: in Section 2, we present the molecular model, the systems studied, and the simulation method for the generation, equilibration, and characterization of the computer-generated athermal mixtures. Section 3 hosts the results of the simulations and the corresponding analysis. Section 4 summarizes the main conclusions and describes current efforts and plans.

## 2. Molecular Model, Systems Studied, and Simulation Method

### 2.1. Molecular Model

In the present work, athermal mixtures of monomers and polymers are simulated. All sites, independent of belonging to a chain or not, are represented as hard spheres of uniform collision diameter (*σ*), which is set as the characteristic length of the system. The pair-wise energy of the hard-sphere (HS) model, *U_HS_*, is given by:

(1) $$U_{HS}\left(r_{ij}\right)=\left\{\begin{array}{c} 0,\;r_{ij}\geq \sigma \\ \;\infty ,\;r_{ij}<\sigma \end{array}\right.$$

where *r_ij_* is the distance between the centers of spheres *i* and *j*. All polymers are represented as linear, freely jointed chains so that bending and torsion angles are allowed to fluctuate freely without any constraints imposed by corresponding potential functions. Bond lengths are, within a numerical tolerance of 6.5 × 10^−4^, equal to *σ*, so that tangency condition is enforced between successive spheres along the chain backbone.

### 2.2. Systems Studied

Simulations are conducted in a cubic simulation cell, with periodic boundary conditions applied in all three dimensions, effectively corresponding to a bulk, unconstrained system. For non-overlapping spheres, like the ones modeled here, packing density (volume fraction), *φ*, is defined as the total volume occupied by the hard spheres, *V_HS_*, over the volume of the simulation cell, *V_cell_*:

(2) $$\varphi =\frac{V_{HS}}{V_{cell}}=\frac{\pi }{6}\frac{N_{at}}{V_{cell\;}}\sigma^{3}$$

where *N_at_* is the total number of hard spheres, independent of being part of chains or not. All systems are composed of *N_at_* = 1200, which are split into *N_pol_* spheres belonging to chains, and *N_mon_* spheres existing as individual entities, so that *N_at_* = *N_pol_* + *N_mon_*. While spheres change identity due to the application of specific MC moves, as will be described in the next section, the populations *N_at_*, *N_pol_*, and *N_mon_* remain constant throughout a given simulation. The *N_pol_* spheres are further distributed into *N_ch_* chains, with an average length (measured in the number of spheres), *N_av_*. Based on the above, the relative number fraction, *x*, (or polymer fraction) can be defined as:

(3) $$x=\frac{N_{pol}}{N_{at}}=\frac{N_{av}N_{ch}}{N_{mon}+N_{av}N_{ch}}$$

The limiting values of *x* = 0 and 1 correspond to the pure monomeric (*N_pol_* = 0; *N_mon_* = *N_at_*) and polymeric (*N_pol_* = *N_at_*; *N_mon_* = 0) HS systems, respectively. In the continuation, we will use also the sub-indices “*mon*” (*x* = 0) and “*pol*” (*x* = 1) to identify them.

The pure-component systems serve further as references to compare against since their phase behavior is well established in the literature [16,17,55,56,58], as described in detail in the introduction. In all simulations considered here, the average chain length is *N_av_* = 12, and chain lengths, *N*, can fluctuate uniformly in the interval $N\;\in \left[6,18\right]$. Table 1 summarizes all mixture compositions simulated in this work. In addition, packing fraction has been explored in the range $\varphi \;\in \left[0.55,\;0.57\right]$, with a step of 0.0025. As explained in the introduction, this interval has been selected to correspond to the regime slightly above the melting point of monomeric (*x* = 0) hard spheres ($\varphi_{mon}^{M}\approx 0.5431$) and below the most recent estimate for the melting point for HS polymers ($\varphi_{pol}^{M}\approx 0.57$), as identified in [58].

### 2.3. Simulation Method

**Figure 1:**
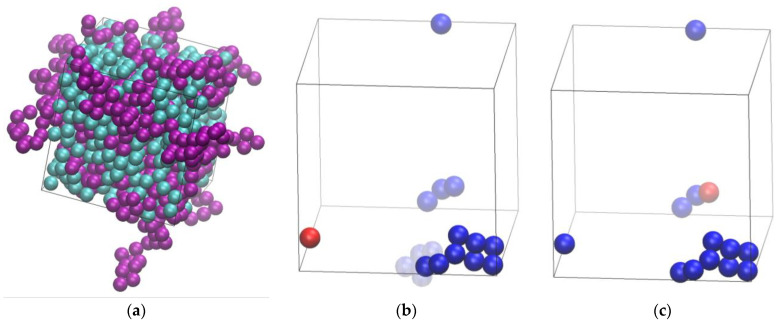
Illustration of the practical implementation of the IdEx1 move. Panel (**a**): system snapshot corresponding to an athermal mixture of *x* = 0.50 at *φ* = 0.56 (*N_at_* = 1200, *N_ch_* = 50, *N_av_* = 12). Individual spheres and ones belonging to polymers are colored in cyan and purple colors, respectively. Spheres belonging to polymers are shown with the coordinates of their centers being fully unwrapped in space. Panels (**b**,**c**) host the states before and after the application of the IdEx1 move, respectively, showing only the involved chain (in blue color) and monomer (in red color). Solid and transparent representations correspond to chain coordinates being subjected to periodic boundary conditions and being fully unwrapped in space, respectively. Image panels were created with the VMD software (version 1.9.3) [73].

All simulations, including the generation and equilibration of the systems, and their successive structural identification, have been performed through the Simu-D suite [54]. The simulator part, as activated for mixtures, is effectively a Monte Carlo (MC) collection of algorithms, which is composed of the following types of moves: (i) monomer displacement, (ii) reptation, (iii) rotation, (iv) flip, (v) intermolecular reptation [48,49], (vi) end-group rearrangement (originally termed CCB [70,71,72]), (vii) simplified end-bridging (sEB) [51], (viii) simplified intramolecular end-bridging (sIEB) [51], and (ix) identity-exchange type 1 (IdEx1) [54]. Moves (ii)–(vi) affect chains by reconstructing a single sphere or a group of spheres, while moves (vii)–(viii) change the connectivity of the chains by deleting and reconstructing bonds without displacing spheres [51,53,54] and are inspired by analogous moves for the simulation of polymer melts in atomistic detail [46,47,49]. Monomer displacement targets individual spheres with the amplitude of the displacement being autocorrected to provide an acceptance rate for the move close to a pre-set value. Finally, IdEx1 changes the identity of a pair of spheres, one being individual and the other belonging to a polymer. A distance condition must be fulfilled to initiate the move: an individual monomer should lie within a bridgeable distance to a chain end. Bridgeable distance corresponds practically to a distance that can be covered by a bond length (further equal to *σ* in the present work since tangency is enforced between bonded spheres). The individual monomer becomes the new chain end while at the other end of the chain the terminal bond is deleted and the sphere at the chain end becomes an individual monomer, maintaining the populations *N_mon_* and *N_pol_* constant. This move, as can be seen in the sketch of Figure 3 in Ref. [54], does not entail sphere displacement(s), but rather proceeds through the deletion and formation of properly selected bonds. Based on this, IdEx1 is expected to have an enhanced acceptance rate at a very high concentration. A practical implementation of the IdEx1 move can be seen in the panels of Figure 1 for an athermal mixture with *x* = 0.50 at *φ* = 0.56.

The attempt probabilities for each move depend heavily on the relative molar fraction. Clearly, in the limit of *x* = 0, only monomer displacement (i) is activated, and in the limit of *x* = 1, moves (i) and (ix) are excluded. All localized polymer moves (ii)–(vi) are executed in a configurational bias way with the number of trials being set at *n_trials_* = 50 at all volume fractions studied here.

Initial configurations are borrowed from Refs. [58,74] corresponding to the pure polymer systems (*x* = 1) of freely jointed chains (*N_ch_* = 100, *N_at_* = *N_pol_* = 1200) at a packing density of *φ* = 0.50. Depending on the target mixture composition, *x*, a matching number of chains is selected at random, and all corresponding bonds are eliminated. Constant-volume simulations are then conducted at *φ* = 0.50 so that each system loses memory of its initial configuration. Then, each configuration is isotropically compressed until the desired density is reached. Finally, production simulations are executed under constant volume, further fixing *N_at_*, *N_pol_*, and *N_mon_*. The presence of sEB (vii) and intermolecular reptation (v) in the MC scheme leads to a (uniform) distribution in chain lengths, which is controlled by properly selecting the spectrum of chemical potentials, as explained originally in Ref. [46] and in the Appendix of Ref. [51]. All simulations are concluded when between 6 × 10^11^ and 8 × 10^11^ MC steps are reached, a duration which is determined to be at least one order of magnitude longer than the time (measured in MC steps) when crystallization occurs. System configurations (frames) are recorded every 10^7^ MC steps. The reproducibility of the results for the pure monomer system (*x* = 0) has been verified by conducting collision-driven MD simulations on the same initial configurations through a simple event-driven algorithm [75] as the one utilized in [67,68].

### 2.4. Post-Simulation Analysis

An essential element of the present modeling scheme is the identification of a possible phase transition and the characterization of the stable morphology at the end of the simulation. Towards this, the characteristic crystallographic element (CCE) norm [76,77] is employed, as implemented in the descriptor part of the Simu-D suite [54]. The CCE norm descriptor relies on the characteristic crystallographic elements and actions, which are uniquely defined for each crystal [78,79,80] to gauge the local structure of computer-generated system configurations made of atoms or particles in three [55,59,63,81] or two dimensions [81,82], in the bulk, and/or on flat surfaces [83,84]. This is achieved through the quantification of the radial and orientational deviation of the “real” environment compared to the ideal one of a given perfect crystal. The lower the value of the CCE norm, the higher the similarity to the reference crystal. By construction, the CCE norm is highly discriminatory, and no two crystals can have simultaneously very low CCE norm values. The exact details of the CCE norm algorithm can be found in Refs. [54,76,77]. Here the hexagonal close-packed (HCP), face-centered cubic (FCC), body-centered cubic (BCC), and simple hexagonal (HEX) crystals are used as reference templates along with the fivefold (FIV) local symmetry. The CCE norm threshold utilized to label a sphere as of *X*-type similarity (*X* corresponding to HCP, FCC, BCC, HEX, or FIV) is set to a value of *ε*^thres^ = 0.245, in consistency with our past studies on athermal packings [55,56,59,67,81,82,83]. Any site not possessing one of the reference similarities stated above (HCP, FCC, BCC, HEX, or FIV) is labeled as amorphous (AMO).

In practice, the CCE norm is applied first by identifying the closest neighbors around each reference site, *i*, through a Voronoi tessellation performed by the *voro++* software (version 0.4.6) [85]. Once the information about the nearest neighbors is available, the crystallographic elements and actions are applied by mapping the computer-generated local environment into one of the ideal reference crystals, *X*, to calculate the corresponding CCE norm, $\varepsilon_{i}^{X}$. The procedure is repeated until all spheres of a system configuration (frame) are analyzed and an order parameter, *S^X^*, is calculated for each crystal, effectively counting the population of sites with such structural similarity:

(4) $$S^{X}=\int_{0}^{\varepsilon^{thres}}P(\varepsilon^{X})d\varepsilon^{X},\;\tau^{c}=\sum_{X}S^{X}$$

where *P*(*ε^X^*) is the probability function of the CCE norm for reference crystal *X*. Based on the above, total crystallinity, *τ^c^*, can be defined as the sum of all crystal-related CCE-order parameters. Given that the studied systems are mixtures of different components, one can further define the corresponding order parameter and crystallinity at the level of polymer (“*pol*”), monomer (“*mon*”), and total (“*tot*”) population according to:

(5) $$S_{tot}^{X}={xS}_{pol}^{X}+(1-x)S_{mon}^{X}\;\tau_{tot}^{c}={x\tau }_{pol}^{c}+(1-x){S\tau }_{mon}^{c}$$

Having available the information from the Voronoi tessellation, the corresponding polyhedron can be reconstructed and its size and shape can be calculated following the concept of Ref. [63]. This is done by considering the Voronoi cell as a body composed of the vertices, all being treated as unit masses. Asphericity, *b*, acylindricity, *c*, and relative shape anisotropy, *k*^2^, can be calculated by the eigenvalues ($\lambda_{1}^{2}$, $\lambda_{2}^{2}$, $\lambda_{3}^{2}$ with $\lambda_{1}^{2}\geq \;\lambda_{2}^{2}\;{\geq \;\lambda }_{3}^{2}$) of the number-averaged analog of the moment of inertia tensor of the Voronoi polyhedron as [63]:

(6) $$\begin{array}{c} b=\lambda_{1}^{2}-\frac{1}{2}\left(\lambda_{2}^{2}+\lambda_{3}^{2}\right)\\ c=\lambda_{2}^{2}-\lambda_{3}^{2}\\ k^{2}=\frac{3}{2}\frac{\lambda_{1}^{4}+\lambda_{2}^{4}+\lambda_{3}^{4}}{{\left(\lambda_{1}^{2}+\lambda_{2}^{2}+\lambda_{3}^{2}\right)}^{2}}-\frac{1}{2} \end{array}$$

The calculation of the above quantities allows us to compare the symmetry and isotropy of the local environment not only between the amorphous and the ordered states but also between individual spheres and those belonging to chains.

## 3. Results

### 3.1. Phase Behavior

**Figure 2:**
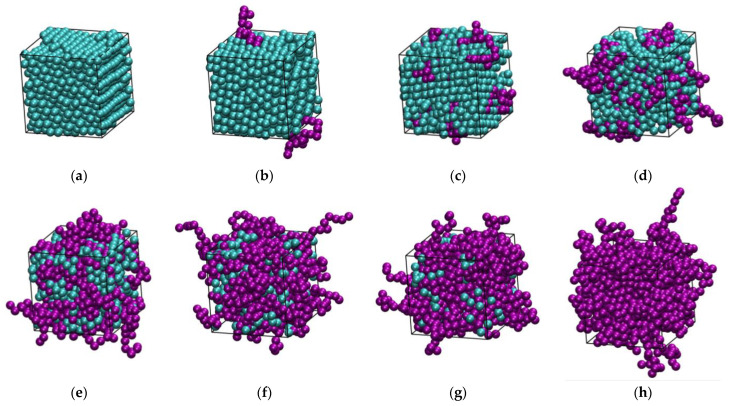
Snapshots at the end of the MC simulation for athermal mixtures at *φ* = 0.5575 and varied number fractions: *x* = 0 (**a**), 0.1 (**b**), 0.2 (**c**), 0.4 (**d**), 0.6 (**e**), 0.8 (**f**), 0.9 (**g**), and 1 (**h**). Individual spheres and those belonging to chains are colored cyan and purple, respectively. The centers of chain spheres are shown with their coordinates fully unwrapped in space. The snapshots were created with the VMD software [73]. The individual panels are also available as 3-D, interactive files in the Supplementary Materials (S.M.).

**Figure 3:**
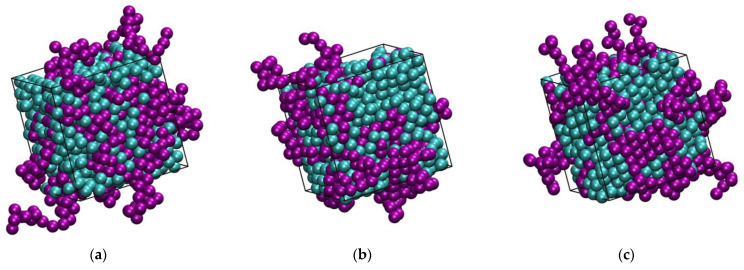
Snapshots at the end of the MC simulation for athermal mixtures of *x* = 0.5 at *φ* = 0.5525 (**a**), 0.5575 (**b**), and 0.5700 (**c**). Individual spheres and those belonging to chains are colored cyan and purple, respectively. The centers of chain spheres are shown with their coordinates fully unwrapped in space. The snapshots were created with the VMD software [73]. The individual panels are also available as 3-D, interactive files in the Supplementary Materials (S.M.).

Figure 2 and Figure 3 host snapshots at the end of the MC simulations for different number fractions, *x*, at a fixed packing density (*φ* = 0.5575) and at various packing densities for a given number fraction (*x* = 0.5), respectively. Individual spheres and those belonging to polymers are colored cyan and purple, respectively, with the latter being shown with the coordinates of their centers fully unwrapped in space.

**Figure 4:**
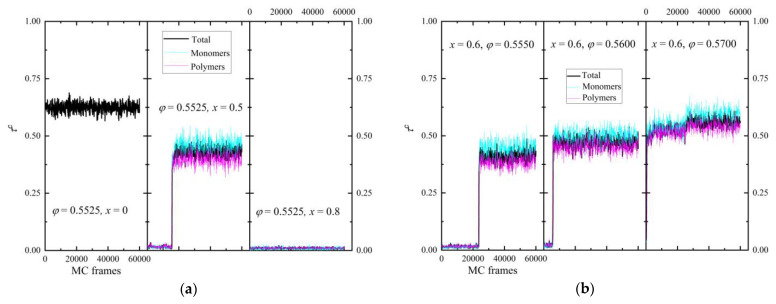
(**a**) Degree of crystallinity of the individual components and of the total population as a function of MC steps for a fixed packing density (*φ* = 0.5525) and varied relative number fraction (*x* = 0, 0.5, and 0.8). (**b**) Same but for a fixed mixture composition (*x* = 0.6) and varied packing density (*φ* = 0.5550, 0.56, and 0.57).

The phase behavior is gauged by tracking the evolution of the degree of crystallinity, *τ^c^*, and of the individual order parameters, *S^X^*, as the simulation evolves, i.e., as a function of MC steps. We should remember here that due to the stochastic nature of the MC scheme and the application of unphysical moves, such as the IdEx1 move (Figure 1)**,** no information related to time is available. Thus, how early or late a phase transition may occur along the MC trajectory is expected to depend strongly on the combination of the *x* and *φ* conditions but cannot be related to a crystallization rate. The evolution of the degree of crystallinity for the individual components and the total population is presented in panels (a) and (b) of Figure 4 for selected systems. In the former, the volume fraction is kept constant (*φ* = 0.5525) and the relative number fraction is varied (*x* = 0, 0.5, and 0.8). In the latter, *x* = 0.60 is fixed and the packing density is systematically changed between the values *φ* = 0.555, 0.56, and 0.57. At *φ* = 0.5525, a concentration value that is above the melting point of individual monomers and quite below that of chains, the purely monomeric system shows a crystallization that occurs immediately so that even the first analyzed frame has already transitioned to the ordered state. As the polymer content increases (*x* = 0.5), crystallization becomes more difficult, as indicated by the transition occurring at later steps and by the lower degree of crystallinity. Finally, at *x* = 0.8, where the polymer component is dominant, no phase transition takes place, and the mixture remains amorphous. In parallel, at a fixed mixture composition (Figure 4b), as packing density increases, so does the crystallinity of both compounds and, accordingly, the total one, and the phase transition occurs sooner.

Two important trends can be further identified with respect to the two components: first, even if the pure components have different melting points, the phase transition occurs simultaneously for both polymers and monomers. This trend is reproducible for all systems studied here that exhibit a phase transition. Second, the degree of ordering for individual spheres is universally higher than the one of spheres belonging to chains. This combination points towards a synchronous crystallization of the whole mixture, which eventually leads to a better-formed ordered environment around an individual site compared to that of a polymer segment.

**Figure 5:**
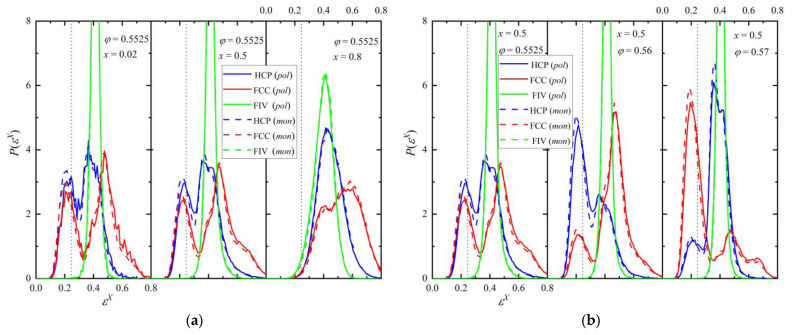
(**a**) Probability distribution function of the HCP (blue), FCC (red), and FIV (green) CCE norm at a fixed packing density (*φ* = 0.5525) and varied relative number fraction (*x* = 0.02, 0.5, and 0.8). (**b**) Same but for a fixed mixture composition (*x* = 0.5) and varied packing density (*φ* = 0.5525, 0.56, and 0.57). The dotted vertical line corresponds to the CCE norm threshold *ε*^thres^ = 0.245.

The distribution of the CCE norm with respect to the HCP and FCC crystals and the FIV local symmetry can provide significant information on the local environment at the final stable phase for each simulation trajectory. In continuation, the following coloring convention is adopted to identify sites in snapshots and curves in figures: HCP (blue), FCC (red), FIV (green), and AMO (yellow). The latter are further represented with reduced dimensions for visual clarity. Figure 5a,b show the probability distribution function for the HCP, FCC, and FIV CCE norm for fixed packing density and relative number fraction, respectively. The vertical dotted line marks the threshold used for the identification of the structural similarity (*ε*^thres^ = 0.245), as explained briefly in the methods section and in more detail in Refs. [76,77]. For a fixed volume fraction (Figure 5a), increasing the polymer content shifts the distribution to the right, leading to a lower crystal similarity. In fact, at *φ* = 0.5525 and *x* = 0.8, the resulting mixture is predominantly amorphous (disordered) and the fivefold population is higher than the combined ones of close-packed crystallites. This is in perfect qualitative agreement with our past findings showcasing the competition between fivefold formation and crystallization for hard-sphere packings [67,68]. For fixed polymer fraction (Figure 5b), the higher the density, the larger the population of sites with crystal similarity. The final ordered morphologies of Figure 5b are also free of fivefold defects. In parallel, a structural competition can be observed between the HCP and FCC crystals, which is not surprising given that thermodynamically they are almost equally stable. Accordingly, based on the CCE distribution data, we expect the emergence of rHCP structures with varied fractions of HCP and FCC segments, without discarding the possibility of purer but still defect-ridden HCP or FCC morphologies.

**Figure 6:**
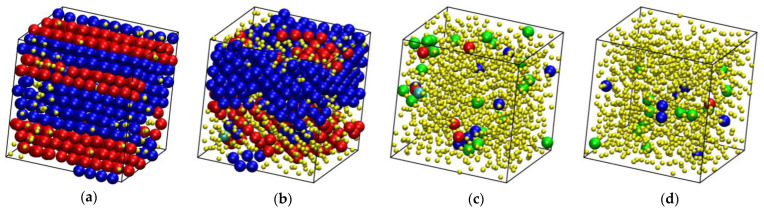
System configurations at the end of the MC simulations at *φ* = 0.5525 and relative number fractions: *x* = 0 (**a**), 0.2 (**b**), 0.8 (**c**), and 1 (**d**). Sites are shown with the coordinates of their centers subjected to periodic boundary conditions and they are colored according to their minimum CCE-norm value: HCP (blue), FCC (red), FIV (green), HEX (purple), and BCC (cyan). Amorphous (AMO) sites are yellow and with reduced dimensions in a 2:5 ratio for visual clarity. The snapshots were created with the VMD software [73]. The individual panels are also available as 3-D, interactive files in the Supplementary Materials (S.M.).

**Figure 7:**
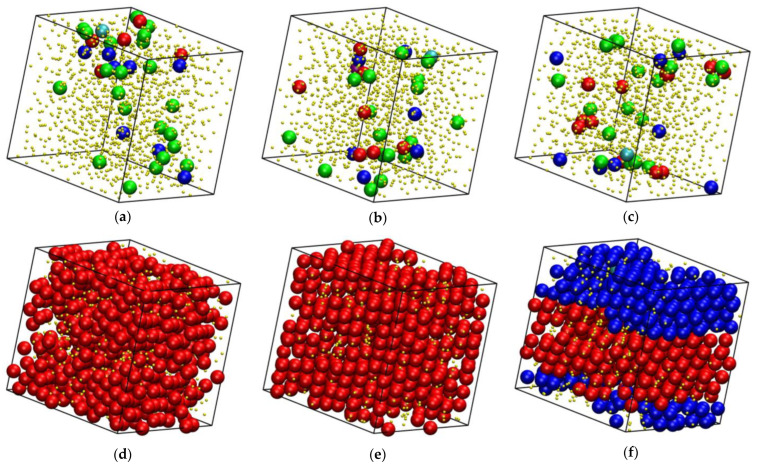
System configurations at the end of the MC simulations for *x* = 0.8 and *φ* = 0.5525 (**a**), 0.555 (**b**), 0.5575 (**c**), 0.565 (**d**), 0.5675 (**e**), and 0.57 (**f**). Sites are shown with the coordinates of their centers subjected to periodic boundary conditions and they are colored according to their minimum CCE-norm value: HCP (blue), FCC (red), FIV (green), HEX (purple), and BCC (cyan). Amorphous (AMO) sites are yellow and with reduced dimensions in a 2:5 ratio for visual clarity. The snapshots have been created with the VMD software [73]. The individual panels are also available as 3-D, interactive files in the Supplementary Materials (S.M.).

Figure 6 and Figure 7 host snapshots at the end of the MC simulation where sites are colored according to the minimum value of the CCE norm and following the color convention described above. Sites with HEX (purple) or BCC (cyan) similarity are very rarely encountered in computer-generated configurations, and when they appear, they correspond to populations that represent less than 1% of the total. Thus, the discussion below focuses exclusively on the closed-packed (HCP and FCC) sites and their antagonist in the form of the FIV local symmetry. In Figure 6, packing density is kept constant (*φ* = 0.5525) and the number fraction is varied (*x* = 0, 0.2, 0.8, and 1), while in the snapshots hosted in Figure 7, the polymer content is fixed (*x* = 0.8) and the volume fraction changes.

Depending on the combination of *φ* and *x*, a wide spectrum of different final structures is obtained, ranging from predominantly amorphous samples, where the fivefold population exceeds that of the ordered sites, to rHCP morphologies formed by HCP and FCC layers of varied thickness and faultiness to even defect-ridden, single FCC morphologies. In this latter case, as can be seen in panels (d) and (e) of Figure 7, the structural defects correspond to disordered sites that lack any kind of similarity to the reference crystals or the fivefold local symmetry. Another clear trend is that the amount of crystal sites increases with increasing packing density (Figure 7) and decreases with increasing polymer content (Figure 6). Furthermore, once the polymer content reaches a critical value, it shifts the melting point to higher packing densities.

**Figure 8:**
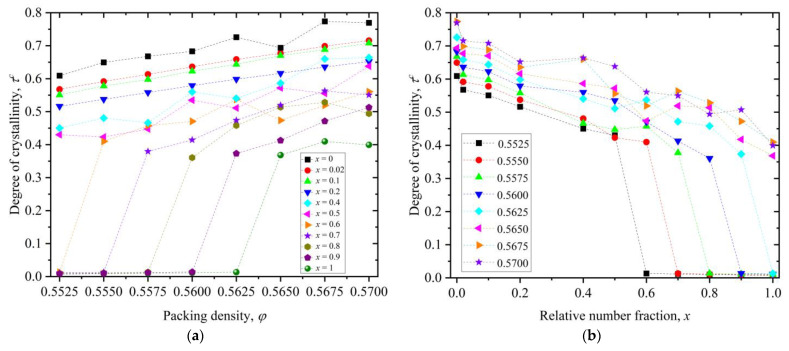
(**a**) Degree of crystallinity, *τ^c^*, as a function of packing density, *φ*, for various relative number fractions, *x*. (**b**) Degree of crystallinity, *τ^c^*, as a function of relative number fraction, *x*, for various packing densities, *φ*. Dashed lines connecting the simulation data points serve only as a guide for the eye.

Based on the results presented above over all simulated systems, a phase diagram can be constructed showing the dependence of the degree of ordering (crystallinity) on packing density for different values of relative number fraction (Figure 8a) and on mixture composition for different values of packing density (Figure 8b).

The melting point is defined here as the lowest observed packing density where the mixture transitions from the initial random packing (of minimal order) to an ordered state where at least 30% of the sites possess a crystal-like local environment. As a first important result of the phase diagram, for the pure polymer system (*x* = 1), a more precise estimation of the melting point of linear, freely jointed chains of tangent hard spheres is obtained, according to which, $\varphi_{pol}^{M}\in (0.5625,\;0.5650]$. The second result applies to the two-component athermal mixture (0 < *x* < 1): the degree of ordering increases, in general, with packing density and decreases with polymer content. More precisely, the pure monomer system (*x* = 0) systematically shows the highest level of ordering, and as polymer content is added, the number of sites with crystal character drops. Even a very low relative number fraction of *x* = 0.02 leads to a significant drop in crystallinity as can be judged by the trends in both panels. Furthermore, it is also clear once the polymer content reaches a critical value the melting point of the mixture is shifted to higher packing densities. For example, adding 10% monomer content to the system (*x* = 0.9) reduces the melting point from $\varphi_{pol}^{M}\in (0.5625,\;0.5650]$ of the pure polymer system (*x* = 1) to $\varphi_{x=0.9}^{M}\in (0.5600,\;0.5625]$.

### 3.2. Polymer Structure

**Figure 9:**
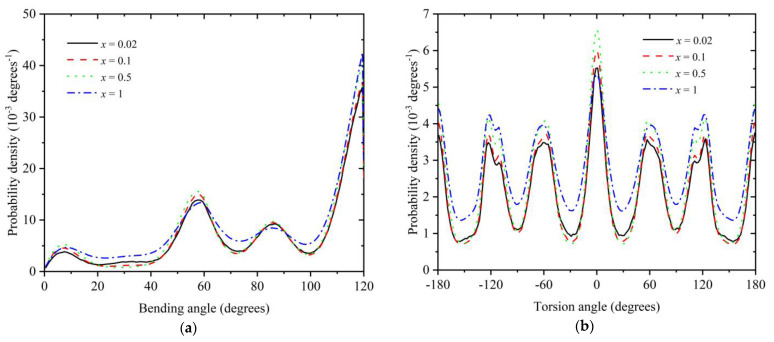
Probability density for the bending (**a**) and torsion (**b**) angles for mixtures with relative number fraction *x* = 0.02, 0.1, 0.5, and 1 at *φ* = 0.57. The scheme of the bond geometry can be found in Figure 1 of Ref [58]: the zero-degrees bending angle corresponds to the straight conformation and the zero-degrees torsion angle corresponds to the all-trans conformation.

In the present section, the local and global structure of polymer chains under various mixture conditions are studied. Figure 9a,b show the bending and torsion angle distributions, respectively, for *x* = 0.02, 0.1, 0.5, and 1 at *φ* = 0.57. According to the data in Figure 8, all systems crystallize, with the degree of crystallinity being approximately *τ^c^* ≈ 0.72, 0.71, 0.64, and 0.40 for *x* = 0.02, 0.1, 0.5, and 1, respectively. Both bending and torsion angle distributions show characteristic maxima (minima) at specific positions along the angle range, all of them being compatible (incompatible) with determined geometric arrangements of the formed polymer crystals, as explained in detail in [56,63]. The shape of the distribution is remarkably similar for all systems except for *x* = 1, where the intensity of the peaks and valleys is reduced. This is because the degree of crystallization is significantly smaller for the pure polymeric system compared to the other mixtures: approximately 60% of the site population possess a highly disordered local environment for *x* = 1, while this percentage drops to around 28–29% for the mixtures with *x* = 0.02 and 0.10. One important conclusion that can be drawn here is that the presence of monomers does not directly affect the local polymer structure but rather influences it indirectly by increasing the degree of order and thus forcing more bending and torsion angles to adopt conformations compatible with the established crystal geometry.

**Figure 10:**
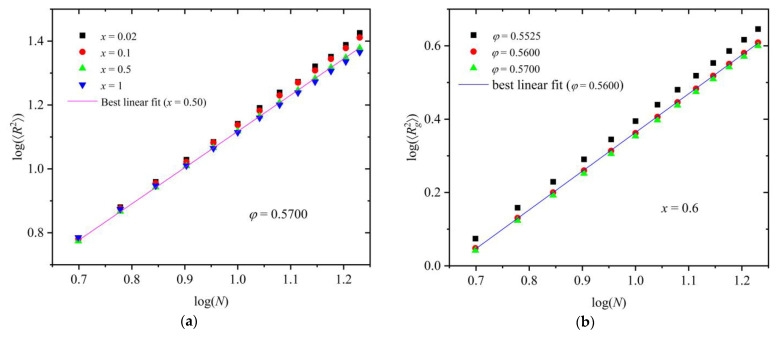
(**a**) Logarithm of mean-square end-to-end distance, log($\langle R^{2}\rangle$), as a function of the logarithm of chain length, log(*N*), at *φ* = 0.57 for *x* = 0.02, 0.1, 0.5, and 1. Also shown is the result of the best linear fit on simulation data for *x* = 0.5. (**b**) Logarithm of the mean-square radius of gyration, log($\langle R_{g}^{2}\rangle$), as a function of log(*N*) for *x* = 0.6 at *φ* = 0.5525, 0.56, and 0.57. Also shown is the result of the best linear fit on simulation data for *φ* = 0.56.

Chain size is typically represented by the mean-square end-to-end distance, $\langle R^{2}\rangle$, and the mean-square radius of gyration, $\langle R_{g}^{2}\rangle$, where brackets denote the average over the number of chains and frames. As in our previous studies [51,64,82], introducing dispersity in chain lengths (here $N\;\in \;\left[6,18\right]$) allows us to study the dependence of chain size on chain length (in the number of monomers) from single simulation trajectories. Figure 10a shows log($\langle R^{2}\rangle$) vs. log(*N*) for various mixture compositions at a fixed packing density of *φ* = 0.57. Figure 10b hosts the log($\langle R_{g}^{2}\rangle$)-vs.-log(*N*) curves at various packing densities for fixed *x* = 0.6. Also shown in both panels are lines corresponding to the best fits of selected simulation data. Through such a fitting, the characteristic Flory exponent, *v* [86], can be calculated, which is equal to *v* = 0.6, 0.59, 0.57, and 0.55 for *x* = 0.02, 0.1, 0.5, and 1, respectively. The observed differences between the mixtures are exceedingly small in the whole composition range. In general, chain size decreases slightly with the polymer content, a trend that can also be related to the reduction in the degree of ordering. Furthermore, for a fixed relative number fraction, the higher the density, the more compact the polymer configurations are. This is expected, as chains tend to coil to minimize the local free volume, while still respecting the geometric constraints of the established crystals.

### 3.3. Homogeneity of the Mixture

Essential information about the structure of an atomic or particulate system can be obtained by the pair radial distribution function, *g*(*r*), which corresponds to the probability of finding a pair of atoms lying apart at a distance, *r*, compared to the analogous probability for a random distribution at the same density [87,88]. The *g*(*r*), here denoted also as *g_tot_*(*r*), is of paramount importance in molecular simulations since it is connected, through a Fourier transform, to the static structure factor, and thus allows for a direct comparison with experiments [75,89,90,91,92]. In parallel, it can be easily calculated from the atomic positions, and it can be used to compute important physical quantities. Compared to an amorphous packing, crystal structure is distinguished in the *g*(*r*) through the emergence of sharp maxima corresponding to specific preferred distances between lattice sites or minima for distances that are not compatible with specific crystal geometries. For molecules, such peaks and valleys are also strongly related to the ones appearing in the distributions associated with the bond geometry, such as, for example, in Figure 9. For binary mixtures of components *a* and *b*, the pair radial distribution function can be calculated for like (*aa*, *bb*) and unlike (*ab*) pair combinations, *g_aa_*(*r*), *g_bb_*(*r*), and *g_ab_*(*r*), providing further information on the structural homogeneity or heterogeneity of the system and on the level of mixing. Here, while all sites correspond to the same molecular description, that of a hard sphere, a distinction can be made between spheres belonging to chains (*a* → *pol*) and individual ones (*b* → *mon*). For monomeric hard spheres, it is now well established that under specific conditions for asymmetric binary mixtures (of small and large sizes or of macromolecules and colloidal particles), phase separation is possible, as documented in theoretical predictions, computer simulations, and experiments [93,94,95,96,97,98,99,100,101,102,103]. Given that polymers and monomers have different melting points, as further demonstrated here through the data in Figure 8, a phase separation should not be excluded, leading eventually to the formation of polymer-rich and monomer-rich regions in the system volume. The homogeneity or heterogeneity of the mixture should thus be reflected in the structural information provided by the *g*(*r*) curves [104,105].

**Figure 11:**
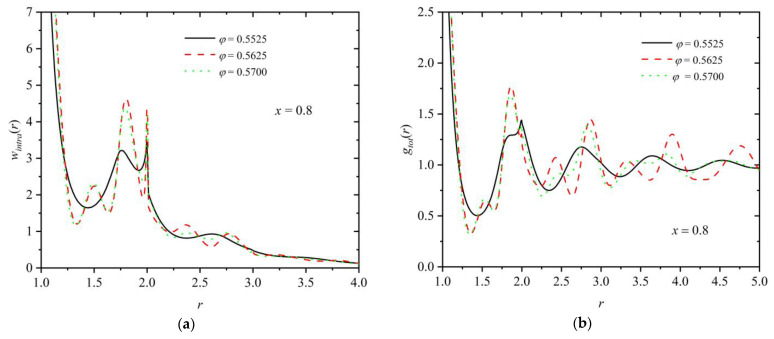
(**a**) Intramolecular pair density function, *w_intra_*(*r*), and (**b**) total pair radial distribution function, *g_tot_*(*r*), as a function of distance, *r*, for a mixture of *x* = 0.8 at *φ* = 0.5525 (*τ^c^* ≈ 0.01), 0.5625 (*τ^c^* ≈ 0.46), and 0.57 (*τ^c^* ≈ 0.49). In the calculation of *g_tot_*(*r*), no distinction is made between individual spheres and those belonging to polymers.

**Figure 12:**
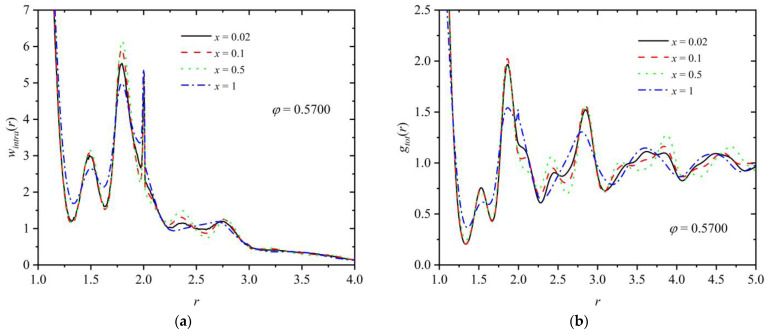
(**a**) Intramolecular pair density function, *w_intra_*(*r*), and (**b**) total pair radial distribution function, *g_tot_*(*r*), as a function of distance, *r*, for a mixture at *φ* = 0.57 and *x* = 0.02 (*τ^c^* ≈ 0.72), 0.1 (*τ^c^* ≈ 0.71), 0.5 (*τ^c^* ≈ 0.64), and 1 (*τ^c^* ≈ 0.64). In the calculation of *g_tot_*(*r*), no distinction is made between individual spheres and those belonging to polymers.

For the polymer component, the intramolecular pair density function, *w_intra_*(*r*), can be further calculated as a structural indicator of the minima and maxima that appear radially along the chain contours. Figure 11a,b present the intramolecular density function and the total pair distribution function, respectively, for a mixture of fixed composition (*x* = 0.8) and at various packing densities (*φ* = 0.5525, 0.5625, and 0.57). Analogous curves are presented in Figure 12 at a fixed packing density (*φ* = 0.57) and varied polymer content (*x* = 0.02, 0.1, 0.5, and 1). First, as expected, the intramolecular chain structure is strongly affected by the degree of the established crystallinity, since the formed ordered morphologies force the local bond geometry to adopt specific arrangements, as also confirmed by the bending and torsion angle distributions in Figure 9. Accordingly, for *x* = 0.8, specific peaks appear for the ordered mixtures at *φ* = 0.5625 and 0.57, which are inherently absent at the lower density (*φ* = 0.5525), where the system remains amorphous. Relative number fraction has no appreciable effect on *w_intra_* for mixtures of similar degrees of crystallinity, as indicated by the data in Figure 12a. The deviations observed for the pure-polymer system can be attributed to the significantly lower degree of ordering compared to the other mixtures. For the calculation of the total pair distribution function (Figure 11b and Figure 12b), no distinction is made between spheres belonging to chains and individual ones. For mixtures that reach comparable ordered states, the characteristic minima and maxima observed in the *g*(*r*) curves have very similar intensities and occur at remarkably close, if not identical, radial distances. Thus, it can be concluded that mixture composition has no direct effect on the global and intramolecular structure of the systems if the same or similar degree of ordering is reached.

**Figure 13:**
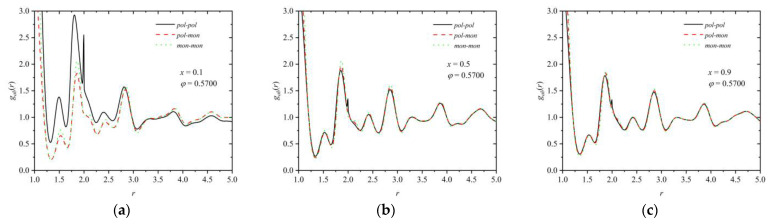
Pair distribution function for like (*pol-pol*, *mon-mon*) and unlike (*pol-mon*) pairs, *g_ab_*(*r*), as a function of distance, *r*, for mixtures of composition *x* = 0.1 (**a**), 0.5 (**b**), and 0.9 (**c**) at *φ* = 0.57.

Distinguishing between spheres belonging to chains and individual ones, the radial distribution function for like (*pol*-*pol*, *mon*-*mon*) and unlike (*pol*-*mon*) pairs can be found in Figure 13 for mixtures of composition *x* = 0.1, 0.5, and 0.9 at *φ* = 0.57.

The data in all three panels clearly demonstrate that there is homogeneity in the mixing of the spheres, independent of where they belong: no chess-like pattern formation or phase separations are observed, which would correspond to the formation of polymer-rich and monomer-rich regions.

### 3.4. Entropic Origins of Crystallization

Given the athermal nature of the mixture, a potential crystallization should be driven by an increase in the total entropy of the system. The entropic origins of the phase transition for hard bodies have been predicted theoretically [7,11] and confirmed through simulations [5,6,12,13,24,65] and experiments [106,107] under various conditions [34,108,109]. For packings of freely jointed chains of hard spheres, constant-volume simulations have demonstrated that the local environment around each site in the crystal phase is more symmetric and more spherical compared to the analogous one in the initial random packing [55,56,59,63,64]. This structural change leads, in turn, to enhanced local mobility of the spheres in the ordered morphology. In MC simulations, this can be quantified by calculating the number of “flippers”, which correspond to chain monomers able to perform local flip moves of small amplitude without causing overlaps with the rest of the spheres, which are held fixed in space. It has thus been documented that the number of flippers increases significantly with crystallization in athermal polymer packings [55,56].

In the present work, the local environment is gauged and compared not only between the amorphous and crystal phases but also between individual spheres and those belonging to chains. Towards this, first, a Voronoi tessellation is performed on the computer-generated configurations through the *voro++* software [85]. This allows the identification of the Voronoi polyhedron around each hard sphere. Then, by considering the Voronoi cell as a collection of point masses located at its vertices, asphericity, *b*, acylindricity, *c*, and relative shape anisotropy, *k*^2^, are calculated through the eigenvalues of the mass moment-of-inertia tensor according to Equation (6).

**Figure 14:**
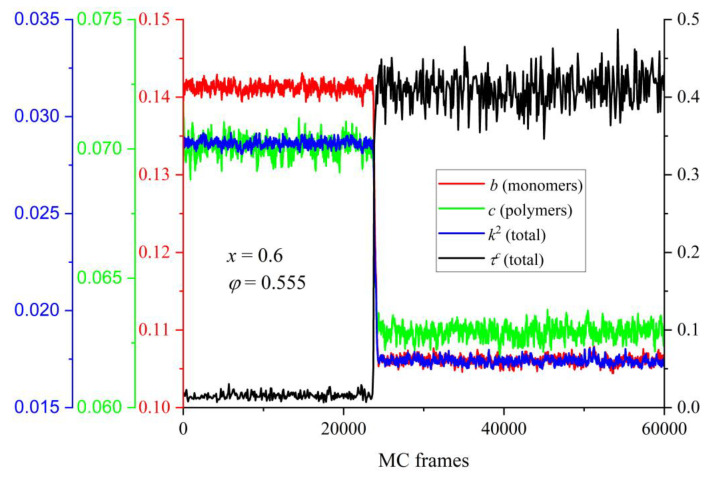
Left axes: exponential running average with a period of 20 for asphericity, *b*, acylindricity, *c*, and relative shape anisotropy, *k*^2^, of the Voronoi polyhedra of individual spheres (red), those belonging to polymers (green), and the total population (blue), respectively, as a function of MC frames. Right axis: the corresponding evolution of the degree of crystallinity, *τ^c^* (total population, black curve). The simulation trajectory corresponds to the athermal mixture with *x* = 0.6 at *φ* = 0.555.

The evolution of the shape measures (*b*, *c*, and *k*^2^) as a function of MC steps is presented in Figure 14 for the mixture of *x* = 0.6 at *φ* = 0.555. Also shown for comparison is the corresponding trend of the degree of crystallinity. There is a very strong correlation between the observed phase transition and the change in the average shape of the Voronoi polyhedra: as *τ^c^* increases very sharply, indicating that the initially amorphous mixture crystallizes, all shape measures instantly adopt significantly lower values. This trend of the shape measures unmistakably implies that the local environment around each site becomes more spherical, symmetric, and isotropic. Additionally, this shift occurs simultaneously with crystallization and affects the total population. The structural change in the local environment, as reported here, is in perfect agreement with past simulation findings on the pure polymer [55,56] and monomer [67,68] systems. Quantifying the transition of the shape of the Voronoi cells for the specific mixture of *x* = 0.6 at *φ* = 0.555, *b*, *c*, and *k*^2^ are reduced by 33.1, 13.6%, and 66.8% for individual spheres and by 31.7, 11.4 and 63.2% for spheres belonging to chains, respectively. Clearly, among the three measures utilized here, the structural alteration in the average polyhedron, as imposed by crystallization, appears most reflected in the relative shape anisotropy.

**Figure 15:**
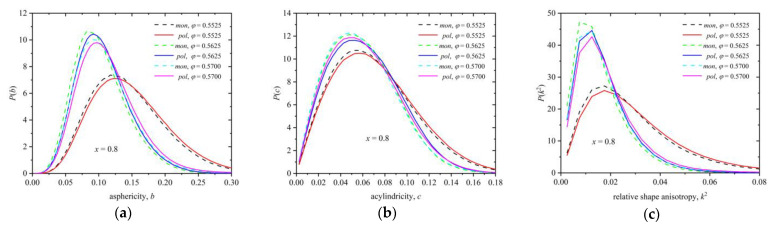
Probability distribution function for asphericity, *b* (panel (**a**)), acylindricity, *c* (panel (**b**)), and relative shape anisotropy, *k*^2^ (panel (**c**)), for the mixture with composition *x* = 0.8 at *φ* = 0.5525 (*τ^c^* ≈ 0.01), 0.5625 (*τ^c^* ≈ 0.46), and 0.57 (*τ^c^* ≈ 0.49). Dashed and solid lines correspond to individual monomers and those belonging to chains, respectively.

Figure 15 presents the probability distribution function of all three shape measures for the Voronoi polyhedra of spheres belonging to chains (solid lines) and of individual spheres (dashed lines) for the mixture of *x* = 0.8 at *φ* = 0.5525, 0.5625, and 0.57. The distributions are obtained by considering the final, stable part of the MC trajectory, which shows the following degree of crystallinity: *τ^c^* = 0.01 (*φ* = 0.5525), 0.46 (*φ* = 0.5625), and 0.49 (*φ* = 0.57). Similarly, Figure S1 (see Supplementary Materials, S.M.) shows the shape measures, this time over all spheres independent of being individual or part of chains, at *φ* = 0.57 for *x* = 0.02, 0.1, 0.5, and 1. The higher the degree of local order, the higher the symmetry, isotropy, and sphericity of the local environment. Between individual spheres and those belonging to chains, while the difference is small, the local environment around individual spheres is systematically more spherical and symmetric.

**Figure 16:**
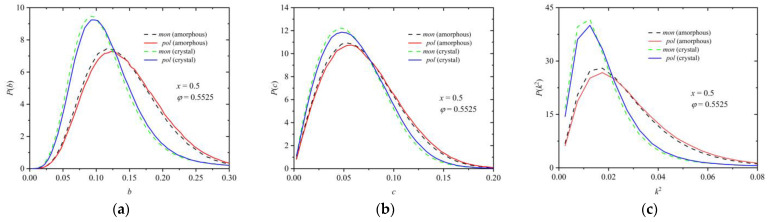
Probability distribution function for asphericity, *b* (panel (**a**)), acylindricity, *c* (panel (**b**)), and relative shape anisotropy, *k*^2^ (panel (**c**)), for spheres belonging to chains (solid lines) and individual ones (dashed lines) in the initial random and the final crystal phase for the mixture with composition *x* = 0.5 at a packing density *φ* = 0.5525.

Similar conclusions can be drawn from the data in Figure 16, which correspond to a mixture of composition *x* = 0.5 at a packing density of *φ* = 0.5525 with the distributions of the shape measures being calculated in the initial random and the final ordered phases. An essential difference can be observed when the Voronoi cell is compared between the two phases: in the crystal morphologies, the distributions for all three shape measures shift significantly closer to zero and their peaks become sharper. Thus, because of the phase transition (crystallization), the local environment around each site becomes more spherical and symmetric. Between spheres belonging to chains and individual ones, the difference is again small but clearly systematic: the individual spheres have “better” crystal environments than the ones belonging to chains. Thus, individual spheres can more efficiently explore their surroundings.

The less symmetric environment observed for spheres as part of polymers can be attributed to the constraints imposed by chain connectivity: as tangency is enforced between bonded spheres, by construction, a sphere belonging to a chain has two (if it is an internal sphere) or one (if it is a chain end) neighbor(s) that lie in a distance equal to the sphere diameter. For the local environment to be perfectly symmetric in radial terms, this would require the remaining 10 (or 11) neighbors to also be tangent. Practically, this would lead to the formation of the most compact crystal (HCP or FCC) with a local density approximately equal to 0.7404. This should not be possible in constant-volume simulations, like the ones presented here, where packing density is set in the range $\varphi \in \left[0.5525,\;0.5700\right]$, much lower than the maximum one achieved for the HCP and FCC crystals, where the 12 closest neighbors are tangent to the reference site. Thus, the bond tangency condition produces a geometric frustration in the formation of the athermal polymer crystal, which is absent in the case of individual monomers. The existence of bond gaps [60] between connected spheres could alleviate such geometric frustration, as explained in [59].

**Figure 17:**
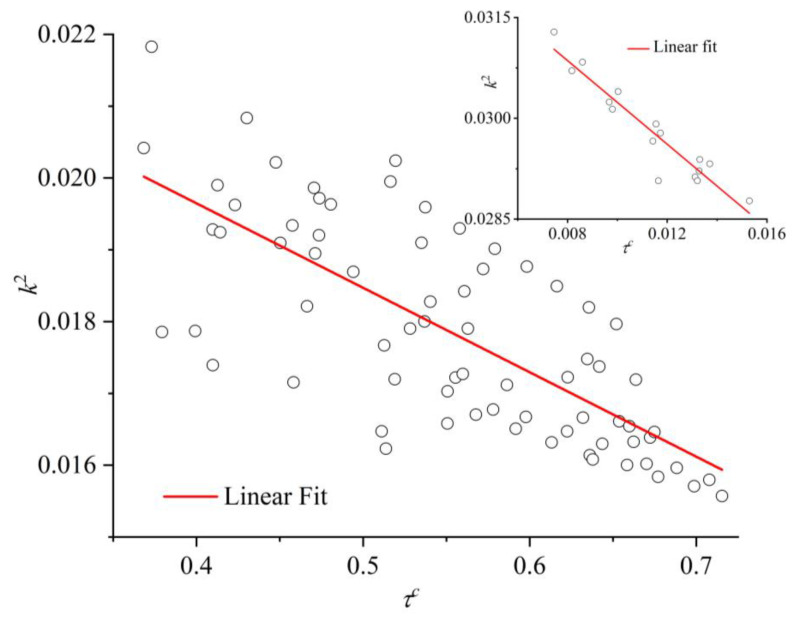
Parity plot of the degree of crystallinity, *τ^c^*, and the relative shape anisotropy of the Voronoi polyhedra, *k*^2^, for the total population in the stable, final part of the MC trajectory for each athermal mixture studied here. Data in the main panel and inset correspond to high-crystallinity (ordered) and low-crystallinity (random) mixtures, respectively. Also shown are the best linear fits (red lines) of the corresponding simulation data.

The correlation between the established degree of crystallinity and the relative shape anisotropy of the Voronoi polyhedra is shown in the parity plot of Figure 17. The data correspond to the total population of the mixture and are obtained from the final, stable part of the MC simulation. The main panel shows the data for the high-crystallinity (ordered) systems, while the inset presents the corresponding data for the low-crystallinity (amorphous) mixtures. Also shown are the best linear fits of available simulation data. The parity plot further demonstrates the strong correlation between the established crystallinity and the isotropy of the local environment.

## 4. Conclusions

We have presented results on the phase behavior of athermal mixtures of hard-sphere polymers and monomers. The combined effect of packing density and composition on the phase behavior of mixtures is systematically studied through extensive MC simulations. The system configurations are initially generated, then equilibrated, and finally characterized through the simulator and descriptor parts of the Simu-D software at volume fractions above the melting point of monomeric hard spheres and below that of fully flexible polymers of tangent hard spheres.

As a first result, we determine the melting point of pure athermal polymers to lie in the interval of $\varphi_{pol}^{M}\in (0.5625,\;0.5650]$, which is a more precise estimate than the previous ones reported in the literature. As polymer content is added, the degree of ordering decreases, and depending on the relative number fraction, the melting point of the mixture can shift to higher packing densities.

The phase transition, when it occurs, is synchronous between the two species, even at packing densities where pure polymer packings do not crystallize, revealing a synergy in the crystallization mechanism. The calculation of the pair radial distribution function and visual inspection of the ordered configurations show homogeneity in mixing in the crystal phase. Accordingly, we observe no phase separation that could split the system into polymer-rich and monomer-rich domains. In general, the local environment around the individual spheres is more symmetric, isotropic, and spherical compared to spheres belonging to polymers. This is a direct consequence of the geometric constraints imposed by the chain connectivity and the corresponding tangency condition. The local environment of the mixture in the crystal phase is significantly more symmetric and isotropic compared to the one in the initial random packing, revealing the entropic origins of the phase transition in accordance with past studies on the pure-component systems. Based on the present findings, it is established that proper tuning of the polymer content can be another parameter to control the phase behavior of athermal hard colloidal mixtures based on polymers.

The present modeling study is currently extended to treat athermal mixtures of semi-flexible polymers and monomers and those of semi-flexible chains of different equilibrium bending angles.

## Figures and Tables

**Table 1:** Composition of the mixtures studied in the present work, where *x* is the relative number fraction and *N_ch_* is the number of chains. In all cases, the average chain length is *N_av_* = 12 and the total number of hard spheres is *N_at_* = 1200.

*N_ch_*	0	2	10	20	40	50	60	70	80	90	100
*x*	0	0.02	0.1	0.2	0.4	0.5	0.6	0.7	0.8	0.9	1

## Data Availability

The data presented in this study are openly available in Zenodo at https://zenodo.org/records/13318555, doi:10.5281/zenodo.13318555.

## Supplementary Materials

The following supporting information can be downloaded at: https://www.mdpi.com/article/10.3390/polym16162311/s1, Figure S1: Distribution of shape measures for mixtures at *φ* = 0.57 and for compositions *x* = 0.02, 0.1, 0.5, and 1; Snapshots of Figure 2, Figure 3, Figure 6 and Figure 7 are also available as standalone, interactive 3-D images (pdf format).
