# Supplementary material for: Entropy-Driven Crystallization of Hard Colloidal Mixtures of Polymers and Monomers
Source: Polymers (Basel). 2024 Aug 15;16(16):2311. doi: 10.3390/polym16162311 (PMC11359749; doi:10.3390/polym16162311)
Supplement: Supplementary file 1 [file polymers-16-02311-s001.zip › polymers-3120903-supplementary.pdf]

Supplementary Materials

Entropy-Driven Crystallization of Hard Colloidal Mixtures of Polymers and Monomers

Olia Bouzid, Daniel Martínez-Fernández, Miguel Herranz and Nikos Ch. Karayiannis *

Institute for Optoelectronic Systems and Microtechnology (ISOM) and Escuela Técnica Superior de Ingenieros Industriales (ETSII), Universidad Politécnica de Madrid (UPM), José Gutierrez Abascal 2, 28006 Madrid, Spain; olia.bouzid@alumnos.upm.es (O.B.); daniel.martinez.fernandez@upm.es (D.M.-F.); miguel.herranzf@upm.es (M.H.)

* Correspondence: n.karayiannis@upm.es; Tel.: +34-910677318

Figure S1 shows the distribution of the shape measures for the mixtures at $\varphi = 0.57$ and for compositions $x = 0.02$, 0.1, 0.5 and 1. In all cases the distributions are calculated over all sites, independent of being individual ones or part of chains.

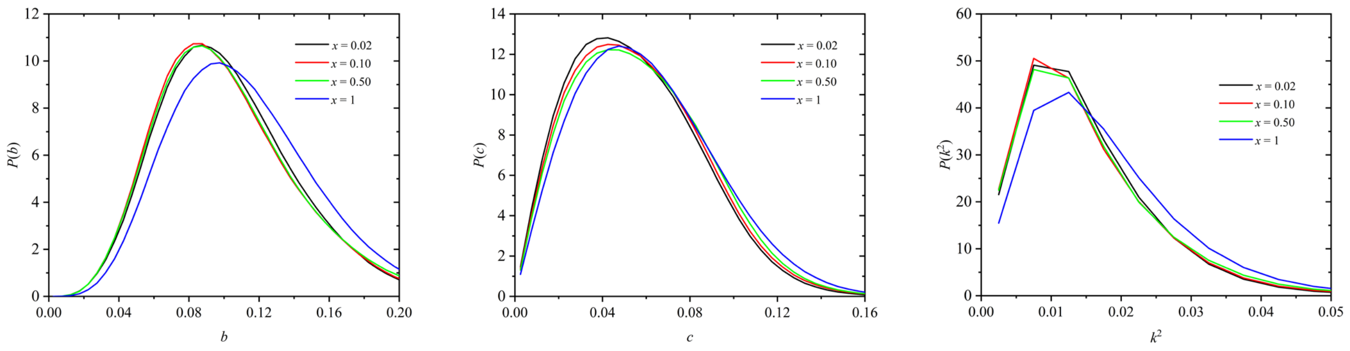

(**a**) (**b**) (**c**)

Figure S1. Probability distribution function for asphericity (**panel a**), acylindricity (**panel b**) and relative shape anisotropy (**panel c**) of the Voronoi polyhedra, as calculated over all spheres in the final, stable part of the MC trajectory at $\varphi = 0.57$. The corresponding degrees of crystallinity are $\tau^c = 0.72$ ($x = 0.02$), 0.71 ($x = 0.1$), 0.64 ($x = 0.5$), and 0.40 ($x = 1$). .
